# Efficacy and safety of levothyroxine (L-T4) replacement on the exercise capability in chronic systolic heart failure patients with subclinical hypothyroidism: Study protocol for a multi-center, open label, randomized, parallel group trial (ThyroHeart-CHF)

**DOI:** 10.1186/s13063-019-3219-5

**Published:** 2019-02-19

**Authors:** Xuan Zhang, Wen-yao Wang, Kuo Zhang, Jian Tian, Ji-lin Zheng, Jing Chen, Shi-min An, Si-yuan Wang, Yu-peng Liu, Yan Zhao, Jing-jia Wang, Min Yang, Yi-da Tang

**Affiliations:** Department of Cardiology, Fuwai Hospital, National Center for Cardiovascular Diseases, Chinese Academy of Medical Sciences and Peking Union Medical College, 167 Beilishi Road, Xicheng District, Beijing, 100037 China

## Abstract

**Background:**

Subclinical hypothyroidism is a common condition in patients with heart failure and is defined as elevated serum thyroid hormone (TSH) with normal circulating free thyroxine (FT4). Evidence on the effect of thyroid hormone treatment is lacking. We designed a randomized controlled trial to compare the efficacy and safety of thyroid hormone supplementation in patients with chronic heart failure complicated with subclinical hypothyroidism.

**Methods/design:**

Eligible participants were identified from the cardiology units of five study centers based on the following criteria: 18 years or older, systolic heart failure with NewYork Heart Association (NYHA) class II–III, left ventricular ejection fraction ≤ 40%, and subclinical hypothyroidism (TSH > 4.78μIU/ml, < 10 μIU/ml + FT4 level within reference range). Eligible patients will be randomly assigned in a 1:1 manner to receive thyroxine replacement therapy plus standard chronic heart failure (CHF) treatment or only standard CHF therapy. Levothyroxine will be administered at an initial dose of 12.5 μg once daily and will be titrated until TSH is within the normal range. The primary endpoints include the difference in distance of the six-minute walk test between 24 weeks and baseline. The secondary endpoints include differences in plasma NT-proBNP levels and serum lipid profiles, changes in the NYHA classification, cardiovascular death, re-hospitalization, differences in echocardiographic and cardiac magnetic resonance imaging measures, and Minnesota Living With Heart Failure Questionnaire (MLHFQ) results between 24 weeks and baseline.

**Discussion:**

ThyroHeart-CHF is designed as a prospective, multi-center, randomized, controlled clinical trial to study the efficacy and safety of thyroid hormone supplementation in patients with chronic heart failure complicated with subclinical hypothyroidism. The study findings will have significant implications for discovering the new therapeutic targets and methods of heart failure.

**Trail Registration:**

ClinicalTrials.gov, NCT03096613. Registered on 30 March 2017.

## Background

Heart failure (HF) is the leading cause of death among cardiovascular diseases, seriously endangering human health and life. The epidemiological characteristics of HF in China are similar to those in developed countries. In 2003, approximately 0.9% of Chinese adults suffered from various degrees of HF. Among subjects of the Framingham Heart Study, the 30-day mortality of HF was 10%, whereas 1-year mortality rates reached 20–30% [1]. In the past two decades, medication for HF has undergone tremendous changes from improving haemodynamics to biomimicry. Despite guidelines and recommended treatments, however, HF mortality has declined but remains at a high level. Therefore, further exploration and discovery of new therapeutic targets and methods for HF are the major issues and challenges we face.

Subclinical hypothyroidism (SCH; elevated TSH with normal circulating free thyroxine (FT3/FT4)), an early stage of clinical hypothyroidism lacking clinical manifestations, has become the new target of treatment for cardiovascular abnormalities. In 2005, we found that hypothyroidism can lead to the occurrence and development of HF even in the absence of heart diseases in animal experiments. The mechanisms of HF caused by hypothyroidism include coronary artery loss, impaired coronary artery flow, and non-adaptive myocardial changes [2]. Growing evidence suggests that hypothyroidism plays a crucial role in the development and progression of chronic HF and is associated with poor prognosis and even death [3, 4].

Epidemiological data have revealed that approximately 13–15% of patients with HF have subclinical hypothyroidism, especially elderly patients [5–8]. Subclinical hypothyroidism (TSH 8.59 ± 4.91 mIU/L) is an independent risk factor for death (hazards ratio = 2.869, 95% confidence interval 1.817–4.532) [9]. A similar conclusion was reported by Iervasi et al. [10] in a study following 3121 patients with heart disease for 32 months. The study also found that subclinical hypothyroidism (TSH 4.5–10 mIU/L) was a predictor of cardiovascular and all-cause mortality [10]. Rodondi et al. [11] reported that subclinical hypothyroidism was an independent risk factor for HF (hazards ratio = 2.33, *P* = 0.03) after 4 years of follow-up of 2555 non-HF patients.

The treatment strategy for HF patients with SCH is intractable given the misconception that SCH is only a simple negative feedback regulation in heart disease and the lack of clinical research on thyroid hormone replacement therapy for HF patients with SCH. American College of Cardiology Foundation (ACCF)/American Heart Association (AHA) guidelines for the management of HF recommended that thyroid function should be routinely assessed in patients with HF [12]. Animal studies and some non-randomized controlled clinical studies have confirmed the beneficial effects of thyroid hormone therapy on cardiac function [13–15]. Therefore, we hypothesized that thyroid hormone supplementation in patients with chronic HF complicated with subclinical hypothyroidism could improve exercise tolerance and quality of life. We use the six-minute walk test (6MWT) to evaluate exercise tolerance, which is particularly useful for measuring the response to medical interventions in patients with moderate to severe heart or lung disease. The 6MWT has also been used to determine the functional status of patients, as well as a predictor of morbidity and mortality [16]. We thus designed this prospective, multi-center, randomized, open labeled, controlled clinical trial to study the efficacy and safety of thyroid hormone supplementation in patients with chronic heart failure complicated with subclinical hypothyroidism.

## Methods/design

### Patient selection

Eligible participants will be identified from the cardiology units of five study centers (Fuwai Hospital, Beijing LuHe Hospital, The Second Hospital of Tianjin Medical University, The First Hospital of Hebe Medical University, Henan Provincial People’s Hospital). All individuals will have their thyroid function checked within 24 h of admission to the hospital or clinical visit. Eligible patients will meet the following inclusion criteria: (1) 18 years or older, (2) systolic HF with NewYork Heart Association (NYHA) class II–III, (3) left ventricular ejection fraction ≤ 40% by echocardiography during screening and randomization, (4) subclinical hypothyroidism (TSH > ULN, < 10 mIU/mL + FT4 level within reference range, (5) receiving standard therapy for chronic heart failure (CHF), reaching target dose or maximum tolerable dose, and (6) provide informed consent. The major exclusion criteria include acute HF or acute exacerbation of chronic HF within the past 2 weeks; scheduled cardiac resynchronization therapy or heart transplantation; history of malignant tumor or life expectancy under 12 months; currently taking medications that may affect thyroid function (LT4, carbimazole, propylthiouracil, amiodarone, lithium); pregnancy and lactation period; participation in another clinical trial within the past 30 days; contraindication or intolerance to evidence-based therapy for CHF, such as beta-blocker, angiotensin-converting enzyme inhibitor, or angiotensin receptor blocker; known hypersensitivity to the trial treatment(s) or diluents (when applicable), including placebo or other comparator drug(s); severe renal dysfunction (estimated glomerular filtration rate ≤ 30 ml/min/1.73 m^2^) or significant hepatic impairment (serum GPT > 120 U/L); and any disorder which, in the opinion of the investigator, might jeopardize a subject’s safety or compliance with the protocol.

Subjects’ participation in this study will be voluntarily and they will sign informed consent forms. No study-associated operation is allowed before written informed consent forms are obtained by the investigators. After informed consent forms are obtained, subject information, including medical history, physical examinations, and adjuvant examinations, will be collected by the investigators to confirm whether the subjects satisfy the inclusion/exclusion criteria. Subjects who conform to all inclusion criteria and do not meet any of the exclusion criteria will be enrolled in this study.

### Randomization

Patients enrolled in this study will receive 2-week standard HF treatment and be invited to have one more thyroid function test 10–14 days after the diagnosis of CHF. Eligible patients who sign written informed consent will be randomly assigned in a 1:1 manner across study centers to receive thyroxine replacement therapy plus standard CHF treatment or only standard CHF therapy. Randomization will be performed using an interactive web response system. Standard treatment for CHF will be administered in accordance with the “2016 ACC/AHA/HFSA focused update on new pharmacological therapy for heart failure” [17]. Levothyroxine will be administered at an initial dose of 12.5 μg once daily and will be titrated until TSH is within the normal range. The 6MWT will be performed at baseline and the 24-week clinical visit.

### Dosage and administration of levothyroxine

**Fig. 1:**
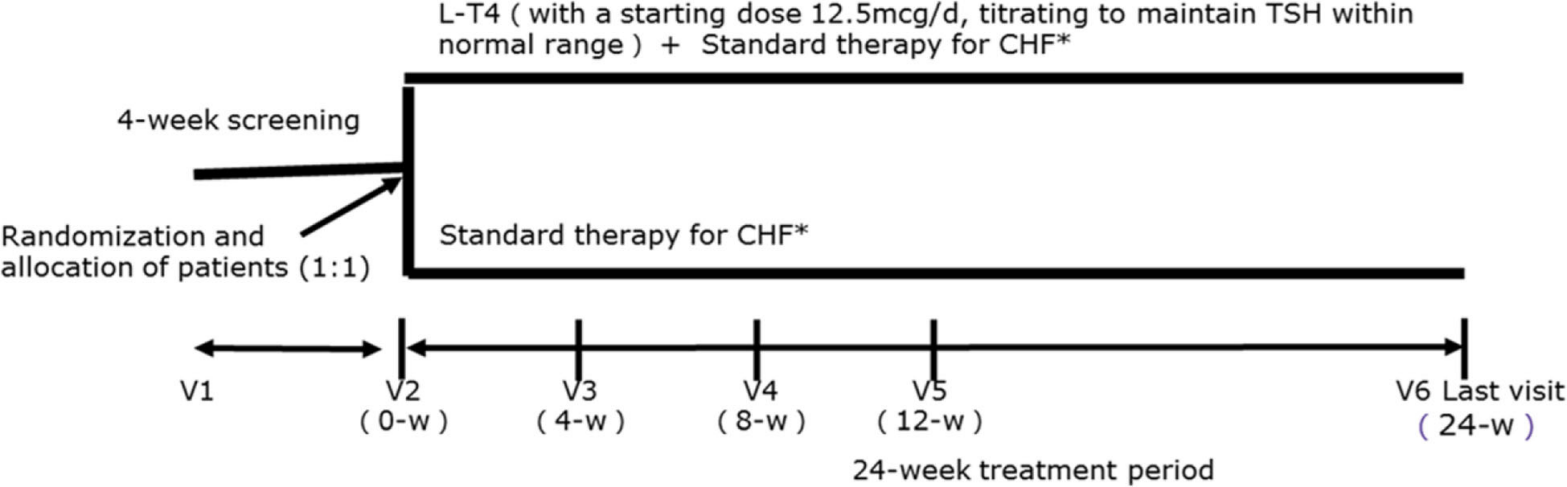
Study Flowchart. *TSH* serum thyroid hormone, *L-T4* levothyroxine, *CHF* chronic heart failure

Levothyroxine will be administered at an initial dose of 12.5 μg once daily and will be titrated until TSH is within the reference range. All the participants will be required to have their thyroid function re-assessed at 4, 8, and 12 weeks to regulate the dosage of levothyroxine, whereas the thyroid status of those in the control arm will be only reviewed and recorded. Investigators will adjust the dosage of levothyroxine primarily based on TSH levels. Specifically, if TSH levels remain above the upper limit of the normal value, 12.5 μg/d of levothyroxine will be added. If TSH levels return to the normal range, the dosage of levothyroxine will be maintained. If TSH levels are below the lower limit of the normal value, the investigator will stop the levothyroxine prescription and thyroid function will be re-assessed 2 weeks later for further intervention (subjects remain in the thyriod hormone replacement therapy (TRT) group with regular thyroid function re-test and corresponding levothyroxine treatment until their TSH levels increase again). Patients who receive levothyroxine but have consistently elevated TSH levels during the 24 weeks will still be considered as “treated” and analyzed in the TRT group. In addition, the percentage of these patients in the TRT group will be calculated and reported, indicating the effectiveness of our conservative titrating strategy for subclinical hypothyroidism. However, these metrics are not the primary or secondary study endpoints. A flow chart of the study is provided in Fig. 1.

### Trial endpoints

#### Primary endpoints

The primary endpoint is the difference in the 6MWT distance between 24 weeks and baseline.

#### Secondary endpoints

- Difference in plasma NT-proBNP levels between 24 weeks and baseline
- Change in NYHA classification at the 24th week
- Percentage of patients experiencing cardiovascular death or HF re-hospitalization
- Percentage of patients experiencing cardiovascular death, re-hospitalization for cardiovascular disease, severe arrhythmia, and stroke
- Difference in echocardiographic and cardiac magnetic resonance imaging measures between 24 weeks and baseline
- Difference in Minnesota Living With Heart Failure Questionnaire (MLHFQ) scores between 24 weeks and baseline
- Difference in serum lipid profiles (total cholesterol, low-density lipoprotein cholesterol) between 24 weeks and baseline

### Follow-up measurements

Clinical follow-up will be performed at weeks 4, 8, 12, and 24 after enrolment. The schedule for visits and evaluation of the subjects is presented in Table 1.

**Table 1:**
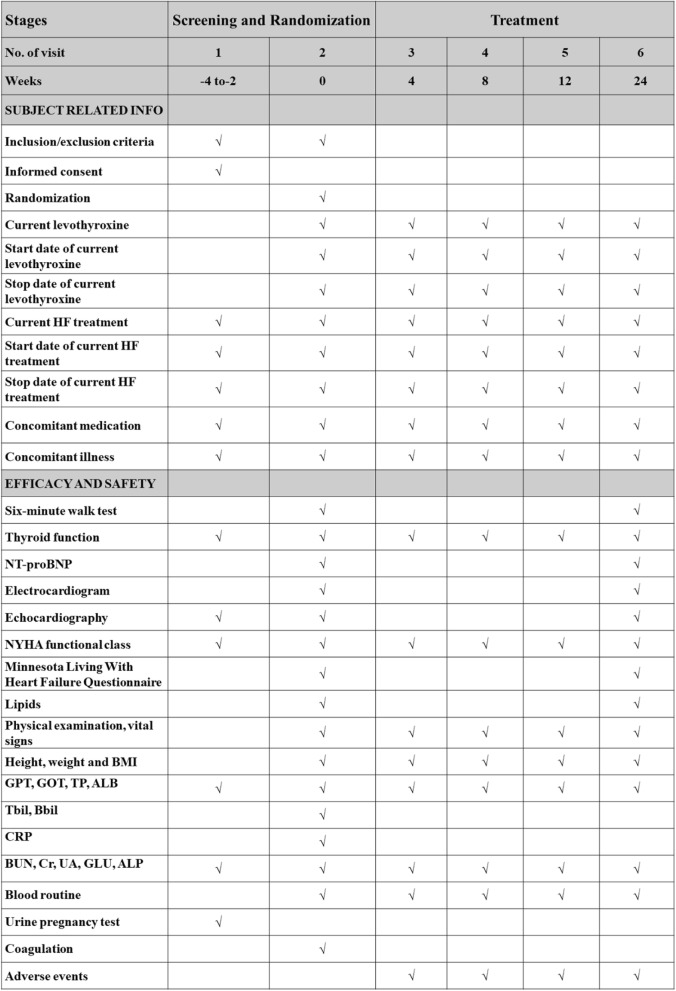
Schedule of assessments

*HF* heart failure, *NT-proBNP* N-terminal pro-brain natriuretic peptide, *NYHA* New York Heart Association, *BMI* body mass index, *GPT* glutamic-pyruvic transaminase, *GOT* glutamic oxaloacetic transaminase, *TP* total protein, *ALB* albumin, *Tbil* total bilirubin, *Dbil* direct bilirubin, *CRP* C-reactive protein, *BUN* blood urea nitrogen, *Cr* creatine, *UA* uric acid, *GLU* glucose, *ALP* alkaline phosphatase

### Data management

An electronic data input system will be used in this study to collect data, and a remote input system will be established according to the electronic case report audited and approved by the sponsors. Subjects’ information required by the protocol should be input by investigators according to the guidelines for completing the electronic case report. The collected data in electronic Case Report Form (eCRF) will be audited and verified by referring to the source document by the clinical research associate of the project during a scheduled visit to ensure data integrity. After data are input into electronic care report tables, quality control and data audit procedures will be performed to ensure the accuracy and reliability of these data. Data managers will audit data deletion, logical error, and other data issues that need to be further explained using computers or artificially. Discovered data issues will be queried or sent to investigators or the Clinical Research Coodinator (CRC) via the electronic data capture system to resolve and update the data in Data Clarification Form (DCF). Investigators or the CRC will manage relevant issues and conduct questions and answers (Q&A) in the electronic data capture system. The updated operation of all data will be audited and preserved in this system. Data issues in the system will be resolved as soon as possible after all subjects complete the study, and the “clean” data will be exported. All data will be audited by the principal investigator, sponsor, data manager, and statisticians, after which the database will be locked.

### Statistics

The Biological Statistics Center of Fuwai Hospital will be responsible for the statistical analysis of the present study. All analyses will be performed based on an intention to treat. Thus, patients will be analyzed in the group to which they were randomized regardless of the treatment they received and whether they deviated from the protocol in any way. All efficacy analyses will be performed on the full analysis set, and safety analysis will be performed on the safety analysis set. The full analysis set includes the set of subjects who are in accordance with the principle of intentionality treatment (ITT principle). Covariance analysis adjusted for traditional risk factors will be used to compare the primary endpoint between two groups, and the absolute difference and 95% confidence interval will be given. In survival analysis, the Kaplan–Meier curve method will be used to calculate the time to clinical endpoints, and the multivariate Cox proportional hazards model will be further applied to estimate the hazard ratios. Continuous variables will be expressed as the mean ± standard deviation. Categorical variables will be described as the frequency or ratio. The normally distributed continuous variables will be compared by using two-sided Student’s *t*-test, and continuous variables that are not normally distributed will be compared using Wilcoxon rank test. Categorical variables will be compared using the two-sided likelihood ratio chi-squared test or Fisher exact test. All statistical analyses will be performed with SAS software, version 9.2 (SAS Institute, Cary, NC, USA).

### Sample size

Sample size and power calculations for the study are based on the following assumptions: two-sided log-rank test; α = 0.05; randomization ratio of 1:1. The sample size calculation is based on the expected change in 6MWT distance at week 24. In another study, the mean difference between groups for the change in 6MWT distance from baseline at week 24 was 40 m with a standard deviation (SD) of 70 m [18]. Based on these assumptions, a sample of 62 subjects per group (124 in total) are required to detect a mean treatment effect of at least 40 m at week 24 with 80% power at a two-sided significance level of 5%, allowing for 20% drop-out.

### Criteria for subject withdrawal

#### Withdrawal from the trial

Participants have the right to withdraw from the study at any time without having to give a reason. The investigator also has the right to withdraw patients from the study drug in the event of inter-current illness, adverse events, serious adverse events, suspected unexpected serious adverse reactions, protocol violations, or administrative or other reasons. Patients who refuse to undergo any form of follow-up are required to leave the study. For study participants who withdraw from the study, an eligible replacement will be recruited if the study is still recruiting.

#### Withdrawal from the medicinal product being investigated

Withdrawal from the medicinal product being investigated means that patients randomized to the intervention group discontinue levothyroxine under the guidance of non-investigators. According to the study, patients will be suspended from levothyroxine supplementation if TSH levels are less than the normal level and/or FT4 is greater than the upper limit of normal; however, this is not a withdrawal from medication. The post-test results will be used to determine whether the patient continues taking levothyroxine. Patients who stop taking levothyroxine are classified as withdrawn from the medication. Regarding adverse reactions during treatment, the clinical event committee will confirm whether the reactions are related to the study drug and intervention medication will be stopped.

#### Premature withdrawal of subjects from the trial

The investigator recognizes the need to terminate the study taking in to consideration a patient’s medical condition. Regarding subjects who do not want to continue the clinical study, the investigator will propose study termination.

#### Definition of completion of trial for subjects

A subject will be considered to have completed the study if he or she completed all assessments and procedures during the follow-up period of 24 weeks for primary endpoint events. All randomized patients will undergo a follow-up assessment (from the randomization to 24th week) to determine exercise capability, survival status, and hospitalization data. If the subject dies during this period, the study coordinator and/or principal investigator should make every effort to obtain the details of the subject’s death from the relevant hospital or physicians and/or relatives. If confirmation of death is not available by these means, the study coordinator and/or principal investigator will attempt to confirm the subject’s death through a death index search (through the Chinese household registration system).

### Participant safety and adverse events

All serious adverse events, including aggravated HF, malignant arrhythmias, myocardial infarction, stroke, severe hepatic and renal dysfunction, sudden death, and hyperthyroidism crises occurring after randomization and before hospital discharge, are to be recorded on the study forms and will be the subject of comparisons between the randomized study treatment groups. Any serious adverse events that are thought by a patient’s own doctors or the study team to be due to the study treatment (i.e., serious adverse reactions) with a reasonable probability are to be reported to the Fuwai trial coordinating center in an expedited fashion.

### Clinical endpoint assessment committee

The safety assessment mainly involves the clinical endpoint assessment committee, which includes three independent internal medicine professors who are not involved in this study. The committee mainly covers the assessment and determination of adverse events.

## Discussion

ThyroHeart-CHF is designed as a prospective, multi-center, randomized, controlled clinical trial (RCT) to study the efficacy and safety of thyroid hormone supplementation in patients with chronic HF complicated with SCH. To the best of our knowledge, this is the first and the largest prospective RCT assessing the effectiveness and safety of thyroid hormone supplementation therapy in HF patients with SCH.

The 6MWT is particularly useful for measuring the response to medical interventions in patients with moderate to severe heart or lung disease. It has also been used as a one-time measure of functional status of patients as well as a predictor of morbidity and mortality [16].

Once researchers have considered the probability that reduction of thyroid hormone levels is the body’s feedback mechanism for self-protection in critically ill conditions, thyroid hormone levels can be lowered to help reduce metabolic rate and thus protect heart function. This lack of understanding is why some researchers do not rectify hypothyroidism in patients with severe disease [19]. In recent years, however, SCH has been shown to increase the risk of HF and all-cause mortality in cohorts. Thus, we considered that hypothyroidism that occurs in the acute stage of severe heart disease might play a role in reducing metabolism and decreasing myocardial oxygen consumption to a certain extent. However, persistent hypothyroidism may aggravate myocardial damage and lead to myocardial cell and heart dysfunction, thus affecting the short- and long-term prognoses of patients with HF. Thus, this neuroendocrine disorder should be corrected.

The mechanisms by which thyroid hormone supplementation improves the outcome of HF patients with SCH, including inhibition of myocardial remodeling and cardiomyocyte apoptosis and improvement of angiogenesis, were validated in animal models [20, 21]. In recent years, researchers have given more attention to myocardial hypothyroidism, which is characterized by normal serum thyroid hormone levels in various heart diseases but a significant decrease in myocardial T3 levels, which indicates that the cardiovascular system is in hypothyroidism [22]. In addition, some researchers have suggested that the degree of myocardial fibrosis and myocardial perfusion/metabolism mismatch may be more serious due to hypothyroidism when HF is combined with hypothyroidism. This situation could lead to severe myocardial damage, cardiac dysfunction, and bad long-term prognosis. We administered thyroid hormone replacement therapy to acute myocardial infarction rats [15]. The results showed that matrix collagen was reduced in the animals in the treated group compared with the control group, and myocardial remodeling was improved, suggesting that the degree of myocardial fibrosis was reduced [15]. Therefore, based on data from previous studies, we also hypothesize that increased myocardial perfusion and reduced myocardial fibrosis can be obtained by supplying thyroid hormones to patients with congestive HF. The Cochrane systematic review of levothyroxine replacement for SCH summarized the evidence from RCTs up to 2006 [23]. It concluded that there was some evidence for improved cardiac function with levothyroxine replacement, but data were available for only 350 patients in 12 RCTs, many of which were of a short duration (range 6–14 months). The Thyroid hormone Replacement for Untreated older adults with Subclinical hypothyroidism trial (TRUST) is the largest RCT to detect clinically worthwhile benefits from levothyroxine replacement for SCH in elderly patients. The mean (±SD) thyrotropin level was 6.40 ± 2.01 mIU per liter at baseline; at 1 year, this level had decreased to 5.48 mIU per liter in the placebo group compared with 3.63 mIU per liter in the levothyroxine group (*P* < 0.001) at a median dose of 50 μg. However, the authors noted that their study was underpowered to detect any effect of levothyroxine on the incidence of cardiovascular events or mortality [24]. Therefore, the TRUST study cannot exclude the possibility that levothyroxine treatment may provide cardiovascular protection. Sato et al. [25] published the first study to demonstrate the association of SCH with adverse prognosis in HF patients. As an observational study without interventions for SCH, this study provides more adequate evidence on the feasibility of the current study. If our hypotheses are supported, the study findings will have significant implications for discovering new therapeutic targets and methods for HF.

### Study limitation

Although we expect that ThyroHeart-CHF will be an important clinical trial, there are certain limitations to its design. The patient population is relatively small and will thusnot allow subgroup analyses, which could be necessary depending on the population included. In addition, all of the subjects included in the study will be Chinese.

Participant recruitment is in progress.
